# Correlations between monitor alarm workload and overall workload in Swedish emergency departments: a prospective multicenter cross-sectional study

**DOI:** 10.1186/s12873-026-01733-2

**Published:** 2026-08-17

**Authors:** Sebastian Johansson, Erika Johannesson, Daniel B. Wilhelms, Jens Wretborn

**Affiliations:** 1 Emergency Department, Region of Jämtland Härjedalen, Östersund, Sweden; 2 Department of Biomedical and Clinical Sciences, Linköping University, Linköping, Sweden; 3 Clinical Department of Emergency Medicine in Linköping, Region Östergötland, Linköping, Sweden

**Keywords:** Clinical alarms, Monitor alarms, Workload, Alarm fatigue, Emergency department

## Abstract

**Background:**

Emergency department staff work under substantial and variable demands. Physiologic monitoring is an integral part of the ED working environment and supports the observation of several patients at the same time, but the alarms generated by monitoring equipment may also add to staff workload. We examined the relation between perceived alarm workload and overall workload and assessed whether operational factors were associated with alarm workload after adjustment for overall workload.

**Methods:**

We conducted a prospective, multicenter, cross sectional study at three Swedish emergency departments. During clinical shifts, staff rated their overall workload and alarm workload during the preceding hour on a scale from 0 to 6. Survey responses were matched by emergency department team and hour with patient data from electronic health records and alarm data from monitoring systems. Pearson correlation was used to assess the relation between the two workload measures. Multivariable linear regression was used to examine associations between alarm workload and prespecified operational variables.

**Results:**

A total of 184 surveys covering 81 h were collected. Alarm workload was moderately correlated with overall workload *r* = 0.53, with *P* < 0.001. After observations without patients were excluded, 68 h were included in the regression analysis. Overall workload, the proportion of patients arriving by ambulance, and the frequency of technical alarms were associated with greater alarm workload in the adjusted analysis. Monitoring equipment was considered helpful in 68% of assessments and disruptive in 18%.

**Conclusions:**

Perceived alarm workload was moderately correlated with overall workload. A higher proportion of patients arriving by ambulance and a higher frequency of technical alarms were associated with greater alarm workload after adjustment for overall workload. These findings may help guide efforts to reduce the workload associated with monitor alarms in emergency departments.

**Supplementary Information:**

The online version contains supplementary material available at 10.1186/s12873-026-01733-2.

## Introduction

Emergency departments (ED) operate under conditions of high patient volume, prioritizing among competing clinical demands, time-critical decisions and tasks [1, 2]. This places substantial workload on staff and increases the risk of diagnostic errors and procedural delay [3, 4], as well as negative effects on staff’s wellbeing and can be associated with burnout [5]. To continuously monitor patients vital signs enable healthcare workers to facilitate the observation of several patients simultaneously, preferably the most critically ill patients. Physiologic monitoring typically includes saturation, heart rate- and rhythm, blood pressure, temperature and respiratory rate and is an integral part of the ED working environment. The monitoring equipment generates alarms when vital-sign thresholds are breached, called *clinical alarms*, or due to equipment malfunction, referred to as *technical alarms*. By design, these alarms should alert clinicians to early signs of patient deterioration or risk of equipment failure [2, 6]. In practice, however, concerns have been raised that the majority of alarms are false, non-actionable, or caused by technical malfunction rather than by genuine physiologic change, and the resulting alarm overload has been linked to a phenomenon termed alarm fatigue [2, 7]. Alarm fatigue is described as the desensitization and delayed response to alarms among health care workers and has been recognized as a patient-safety hazard across hospital settings for more than a decade, and national safety organizations continue to identify alarm management as a priority area for improvement [8–10]. Most of the existing evidence originates from intensive care units and inpatient wards, where intervention studies have demonstrated that customizing alarm thresholds, standardizing alarm-management protocols, and reducing nuisance alarms can improve both staff performance and patient outcomes [10]. The ED, however, differs fundamentally from these settings and whether the workload of alarms is perceived differently from workload in general by ED staff is unknown. Unselected patient populations, open workspaces, rapid patient turnover, frequent overcrowding, and a broad spectrum of clinical acuity create a monitoring environment in which alarm workload may interact with overall workload in ways that are not directly transferable from the inpatient setting. In a recent multicenter mixed-methods study, our group described the alarm frequency and pattern across three Swedish EDs. The study highlighted the challenging working environment in the ED connected to a high alarm frequency, resulting in an alarm burden for ED staff. Qualitative data from interviews suggested that the burden of alarms was not easily separated from the burden of clinical work in general and ED staff showed a perceived interaction between workload and burden of alarms, but was not explored in detail [6]. Yet, this distinction is critical. If alarm burden is simply a reflection of workload, then interventions should target workload itself; if, however, specific alarm-related factors contribute independently, targeted alarm-management strategies may reduce the overall burden on staff without requiring changes to patient flow or staffing.

Both excessive workload and high alarm exposure have the potential, independently, to affect ED staff work environments and impair patient safety by compromising clinical decision-making, increase the risk of adverse events, and erode staff well-being. A better understanding of whether these two factors are correlated, and, if so, possibly identifying additional ED specific variables that contribute to perceived alarm workload beyond overall workload alone, is a prerequisite for designing effective interventions. If modifiable factors such as alarm features or certain patient-flow characteristics amplify alarm workload independently of overall workload, they represent actionable targets for improvement in the ED work environment.

## Methods

### Aim

The aim of this study was to determine the correlation between ED staff’s perceived alarm specific workload and overall workload, and identify whether specific ED operational variables, including patient acuity, mode of arrival, patient age, chief complaint, and alarm type, contribute to perceived alarm workload independently of overall workload.

### Study design and setting

This was a prospective cross-sectional study of ED staff assessment of overall workload and alarm specific workload from monitoring equipment through a survey during clinical shifts with matched patient data from the electronic health record and monitoring alarm data. The study was conducted at three EDs in southeastern Sweden (Region Östergötland): one rural, one urban community, and one academic hospital. The region serves a population of approximately 470,000, with a combined annual ED census of about 125,000 visits.

All three ED´s use the Rapid Emergency Triage and Treatment System (RETTS) for triage and is the most common triage tool in Sweden [11]. The RETTS triage tool classifies acuity on a scale from level 1 to 5, level 1 indicating the highest acuity based on vital signs findings and specific chief complaint questions [12]. The study sites use the same local routine on patient monitoring which states that patients with triage acuity 1 and 2 (indicating high acuity) are to have continuous monitoring of all vitals. All three ED´s use the same monitoring equipment from Philips with X3,MX450 or MX550 models with the same alarm pre-set thresholds settings that trigger alarms. Decisions on adjusting the alarm settings can be made by a physician or a nurse. Alarms are classified in three levels, level 1 (most severe) and 2 are both indicating deviation in patient vital signs or heart arrhythmia, and level 3 alerts staff that the monitoring equipment is not working properly. The alarm levels are separated with different alarm sounds, both in tone and intensity to call ED staff’s attention. Alarm signals are sounding both on the bedside and at a central monitor placed at the team workstation. Level 1 alarms are most intense, high pitch tone and must be confirmed manually at the bedside monitor to stop sounding. Level 2 and 3 will silence automatically if vital signs normalize or technical issues are corrected, but still have to be confirmed to resolve. Details of alarm settings, specifications on monitoring equipment and alarm management at the ED sites have been previously described and published in a study from our research group [6].

### Participants

Study participants were a convenience sample of physicians, nurses and nurse assistants working in the ED. At these study sites, units are formally organized within the ED and entitled as a “ED team”, including those healthcare professions in a 1-1-1 ratio as a typical set up.

### Variables

A Likert scale (0–6) was used to measure staffs’ perception of general and alarm specific workload, ranging from no workload (0) to very high workload [6].The variables of ED specific metrics were selected based on statements from previous interviews in context of another study were participants specifically mentioned *patients with chest pain as a reason for the ED visit* were particularly associated with high alarm frequency, as well as *geriatric patients*, *high total number of patients assigned to the ED team*, *volume of high acuity cases* and *patients arriving by ambulance* [6]. For this study we defined geriatric patients as patients over 65 years and high acuity cases as a triage priority of level 1 or 2 according to RETTS.

Alarms were characterized by type, level and timestamps. To create an overview of types of alarms we categorised them based on the topology described by Ruskin et al. (2015) into two categories: *clinical alarms* which indicate deviant vital signs creating level 1(most severe) or level 2 alarms, and *technical alarms* which were level 3 alarms, caused by malfunction or disturbances reading vital sign measurement affecting analyzing performance in the monitoring equipment itself.

### Data sources

All alarm data from the monitoring equipment at all study sites are routinely saved for 60 days and is available from a central server in a register and was collected in full for the data collection time. Data contained information of every individual alarm during the data collections period on monitor ID/serial number, alarm type, timestamp, alarm confirmation time. To include ED specific metrics we used the electronic health record, Cambio COSMIC, to collect data on; patient way to arrival, number of patients, triage priority, acuity level post triage, timestamps, patient age and ED team assignment. No personal data from individual patients was collected from the health record or the alarm data set.

### Data collection

Workload assessments were done using an online survey through *Webropol* survey tool based on six questions (supplements). The survey was iteratively tested on three ED clinicians with research experience. The survey’s main objective was collecting data on ED staff assessments on the overall workload and alarm specific workload from monitoring equipment the last hour, using the likert scale, furthermore participants were asked if the monitoring equipment had been useful or disturbing during the same hour, as well as demographic questions on which ED and ED team they worked at. The survey was made available between 2024-03-05 to 2024-04-19 at the study sites through a QR-code. Participants were recruited when scanning the QR code during their ED shift. A minimum of one hour of work during the shift was used as an inclusion criteria. Since the questions focused on estimations of the last hour, it was possible to participate multiple times, given it referred to a different hour worked. Exclusion criteria were leadership management or supporting roles at the ED without assigned monitors during the hour of assessments. The authors EJ and SJ were onsite to brief staff, answer questions about the study at 4 convenience days during the data collection period. Data from the central alarm server on all alarms produced at the study sites during the same period as the survey was available were collected, together with gathering of data from the electronic health record.

### Statistical methods

Workload and alarm specific workload assessment data was compiled and stratified by ED, team and hour. Individual monitors were identified by serial number and connected to specific teams at the ED´s. This made it possible to determine the alarm frequency, not only in the ED overall but also on specific ED teams at the time of the assessments. We matched data from the electronic health record by the same time interval. When multiple assessments were collected from the same ED team and within the same hour, a mean score was calculated.

The assessments of overall workload and alarm specific workload were then analyzed using a Pearson R test. Multivariate linear regression analysis was used to identify associations between alarm specific workload, workload and patient-related covariates.

Linear regression was chosen because alarm workload was analyzed as a continuous mean score for each team and hour, similar to previous work by our research group [13]. Patient characteristics were entered as continuous proportions because they described the composition of patients assigned to each team during that hour rather than characteristics of individual patients.

In the regression analysis, workload and the number of patients was treated as continuous variables. Arrivals by ambulance, geriatric patients, high acuity patients and patients with chest pain were calculated as the percentage of total number of patients for each team and treated as continuous variables. Alarms were treated as continuous variables and the logarithm of technical alarms was used as the previous data suggested more exponential association [6]. Assessments were excluded in the regression analysis if there were no patients present at the team during the hour of assessment. No additional covariates were adjusted for. Analysis was done using R, version 4.4.

## Results

**Fig. 1:**
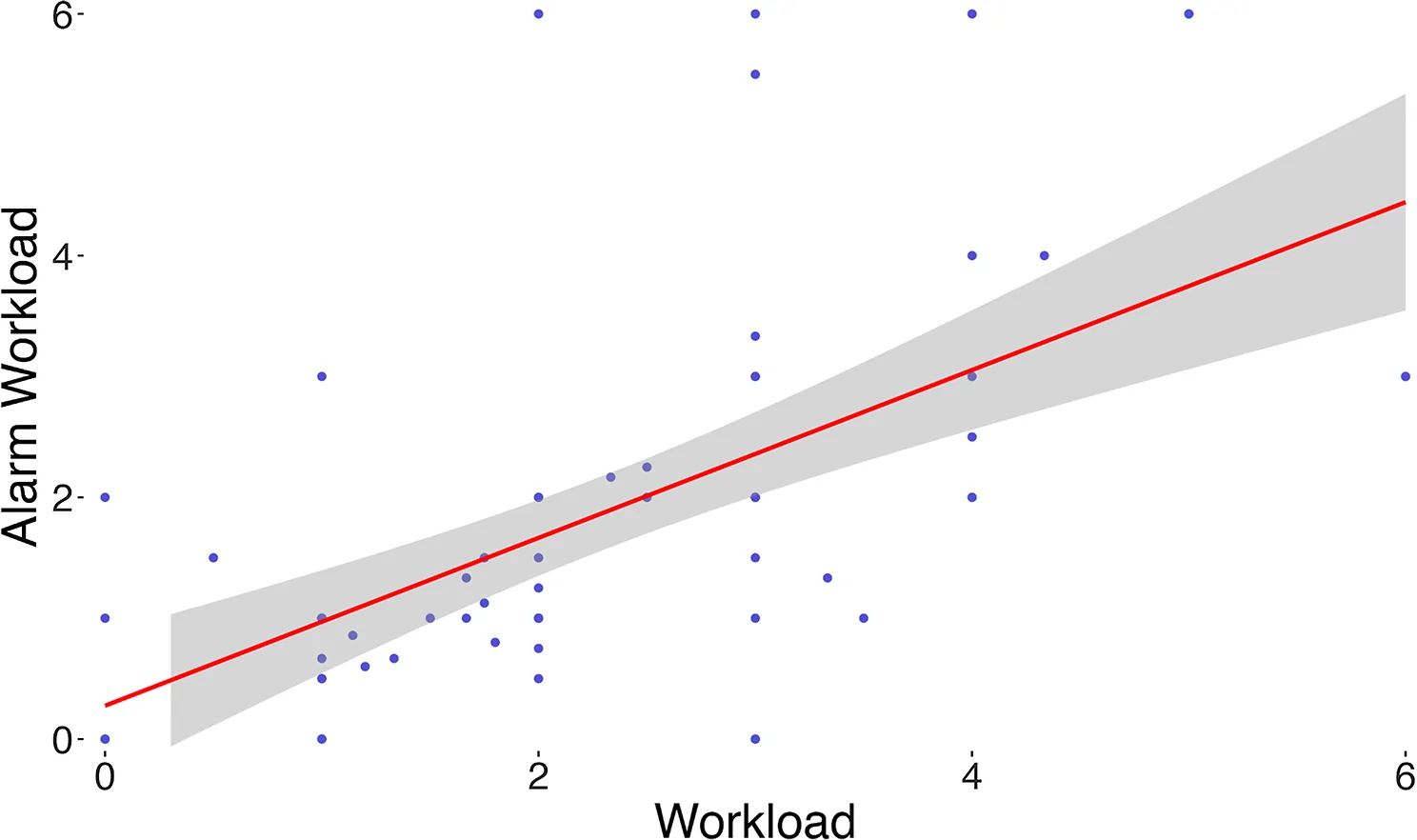
- Scatterplot of correlation between alarm workload and workload

A total of 184 surveys were collected, covering 81 h. Answers were distributed as 30% (*n* = 56) rural hospital, 45% (*n* = 83) academic hospital and 25% (*n* = 45) from the urban community hospital. There was a moderate correlation between alarm workload and workload *r* = 0.53, p-value < 0.001 (Fig. 1).

Almost one fifth (18%) said that they had been disturbed by the monitoring equipment, and 68% said that the monitors had been helpful at the time of assessment.

**Fig. 2:**
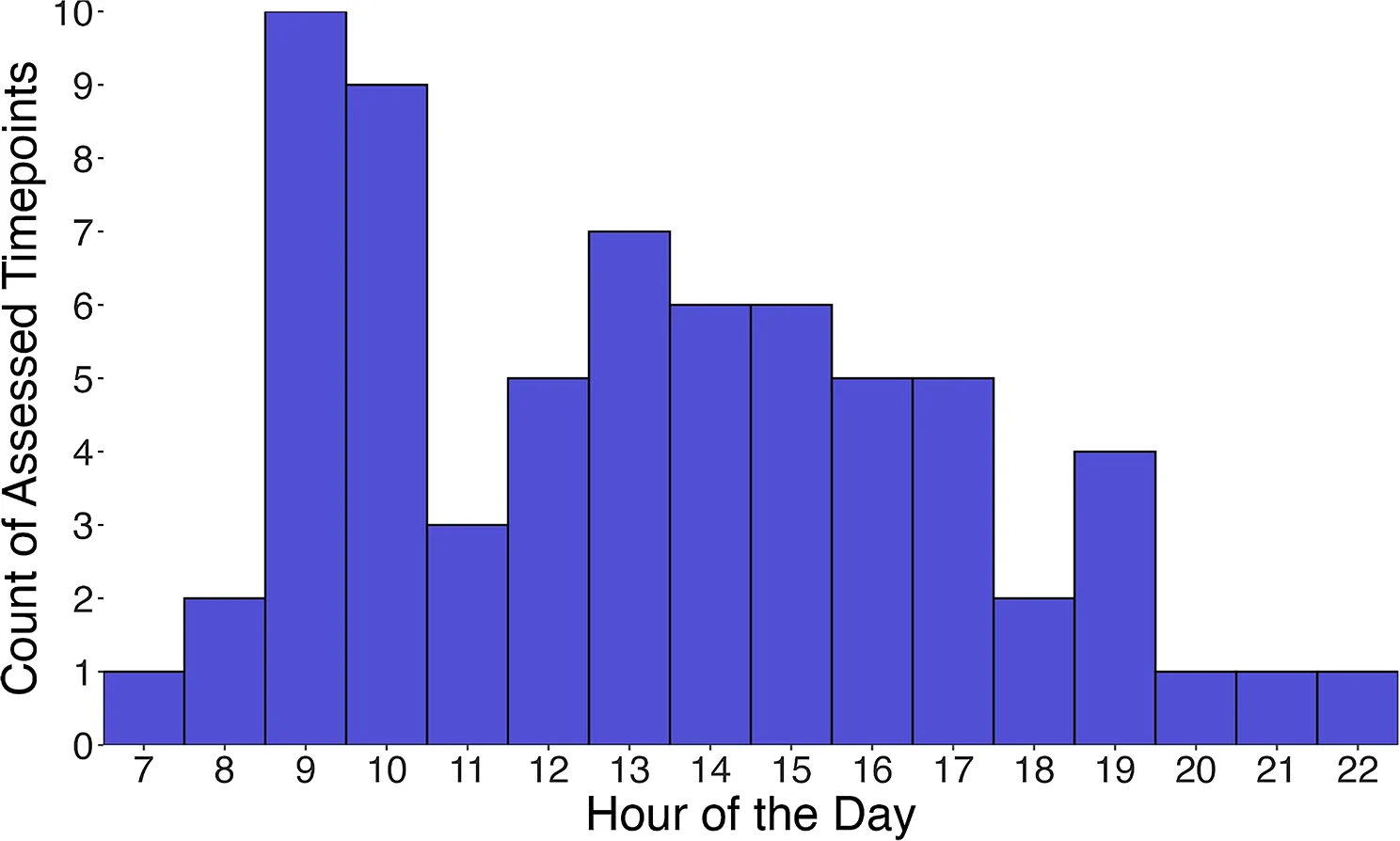
Distribution of number of estimations,* hourly during the day*

The distribution of hours analysed were evenly distributed of the day except for morning hours which were most frequent (Fig. 2).

After exclusions of assessments made by staff where no patients were present, 68 h were included in the analysis. The median workload and alarm workload were 2.0 and 1.4 respectively. On average, the number of patients per team was 5, and technical alarms were nine times more frequent compared to clinical alarms, Table 1. 

When adjusting for potential covariates in the regression analysis, the effect of workload on alarm workload was largely unchanged with a correlation of 0.5 (95% CI 0.3 - 0.8, p<0.001). In addition, there was a positive association between alarm workload and percentage of patients arriving by ambulance (β=1.6, 95%CI 0.4 - 2.9, p=0.01) and technical alarms (β=0.4 95%CI 0.04 - 0.8, p=0.03). High acuity patients, old patients, patients with chest pain and clinical alarms were not associated with increased assessed alarm workload (Table 2)

**Table 1:** summary of workload assessments, patient-related characteristics and alarm data as median (interquartile range) or mean percentage

Characteristic	*N* = 68
Alarm Workload	1.4 (1.00, 2.21)
Workload	2.0 (1.07, 3.00)
Number of Patients	5.0 (2.00, 8.00)
**Patient Characteristics**	
Arrivals by Ambulance	44%
High Acuity Patients	39%
Age over 65	63%
Patients with Chest Pain	13%
Clinical Alarms	2.0 (0.00, 5.00)
Technical Alarms	18.0 (8.00, 27.00)

**Table 2:** Results of the multilinear regression analysis of ED variables

Variable	Correlation (β)	95% Confidence interval	*p*-value
Workload	0.5	0.3–0.8	< 0.001
Patients per team	0.01	-0.08–0.1	0.8
Technical Alarms	0.4	0.04–0.8	0.03
Percentage of patients per team			
- Arrivals by ambulance	1.6	0.4–2.9	0.01
- Acuity 1 or 2	0.5	-0.7–1.6	0.4
- Age > 65 years	-0.7	-2.0–0.6	0.3
- Chestpain	-0.2	-2.1–0.8	0.9

## Limitations

This study has some limitations to be considered when interpreting the results. Data collection was not evenly spread during the data collection period and predominantly done when the authors were on site. This could have affected results considering that data was likely collected from a smaller sample of the individual clinicians that worked on those days and participated in the survey, giving them a disproportionately impact on assessment data and dictating which days and hours to include in the linear regression analysis.

Data were predominantly collected in the morning until lunch and midday, with 70,7% of all assessments collected between 08.00 am and 03.00 pm a timeframe where the total number of patients and alarm frequency typically escalates towards the afternoon and evening. However, in our data 17% of the assessments indicated an alarm workload of ≥ 4, indicating that we manage to collect assessments even when alarm workload and workload were high.

## Discussion

In this prospective cross-sectional study we found a correlation between ED staff’s estimated alarm specific workload from monitoring equipment and overall workload. In addition to workload, the proportions of patients *arriving by ambulance* and the *frequency of technical alarms* seems to affect the ED staff experience of alarm workload. Our findings confirm the close connection of the two variables, with a r-value of 0.53, but also highlights that there are factors that may be amenable to intervention to reduce alarm workload apart from targeting workload. Although the correlation is moderate, meaning that general workload in the ED is influenced by other factors than the burden of monitoring alarms, it plays an important part in efforts to improve the working environment for ED staff.

To our knowledge, this is the first study to quantitatively examine this correlation in the ED setting. Overall workload emerged as the primary driver of alarm workload, aligning with findings from in-hospital and intensive care unit contexts. In a pediatric in-hospital setting it has been shown that high alarm frequency has been associated with increased nursing workload and argued that the escalation in workload from alarming monitoring equipment could be comparable to increased workload as caring for a higher number of patients [14]. In another cross-sectional study of nearly 4,000 hospital nurses found that the majority reported feeling overwhelmed by alarms, which was associated with poorer work environments and prolonged alarm response times. Furthermore, the same study stated that this phenomenon was higher outside the ICU setting and argued that caring for a higher number of patients could impact staff’s experience of alarm workload [15], something that would be applicable to the ED setting.

In this study the number of patients the ED team cared for were not correlated with alarm workload. Interestingly, when we analyzed the number of high acuity patients (level 1 and 2) assigned to the ED team we did not find a significant correlation with alarm workload either, even if the local guideline stated that these patients should always get full continuous monitoring of vital signs and thereby should be more likely to produce alarms. Perhaps our alarm data are affected by the same trends Cvach and colleagues could show in a multicenter study, that a majority of the alarms are produced by a few outlier patients [16], and not necessarily patients of high acuity. An alternative explanation is that lower-acuity patients were also subjected to continuous monitoring, producing alarms perceived as less clinically relevant as the benefit to monitor patients with less acuity may be lower. Alarms from patients with lower acuity may have made alarms from patients with higher acuity more difficult to notice and increased perceived alarm workload across acuity levels. However, alarm fatigue was not measured in this study. Neither this explanation nor the possibility that a small number of patients generated a disproportionate share of alarms could be examined because no patient identifiers were collected under the approved study protocol.

Among all ED visits, 63% were patients over 65 years of age. It’s worth mentioning that this factor was not a significant predictor of increased alarm workload, as it could be easy to make the assumption that this population should be due to a higher comorbidity.

In this study, overall, technical alarms were almost nine times as common, compared to the clinical alarms and was in itself a potentially modifiable factor to alarm workload. We argue that the recommendations of interventions to this point have been too universal, trying to solve all parts of the problem simultaneously and tend to focus on the settings towards clinical alarm thresholds. With the findings in this study, we advocate that technical alarms should be in focus for future interventions when trying to improve ED staff’s alarm workload. Regarding the *number of patients arriving by ambulance* assigned to the ED team, we recommended that ED management and leadership account for the impact of ambulance arrivals on ED staff when planning patient flow and designing local monitoring protocols. A higher volume may increase staff alarm workload and compromise patient safety. However, it is important to highlight that participants in this study also stated that the monitoring of vital signs during the shifts were mostly helpful. There is probably some sort of “sweet-spot” in daily practice, perhaps dependent on individual resilience among ED staff, on when alarm workload risks leading to alarm fatigue.

Future research should aim to explore if the alarm distribution based on different triage levels and reasons for ED visit can be associated with specific alarms, especially technical alarms and if a majority of alarms are produced by a few outlier patients.

## Conclusion

Most respondents considered the monitoring equipment helpful, although some found it disruptive. Perceived alarm workload was moderately correlated with overall workload. In adjusted analyses, a higher proportion of patients arriving by ambulance and a higher frequency of technical alarms were associated with greater perceived alarm workload. These findings may help guide efforts to reduce the workload associated with monitor alarms in emergency departments.

## Supplementary Information

Below is the link to the electronic supplementary material.

Supplementary Material 1

## Data Availability

Aggregate datasets will be made available on reasonable request to the corresponding author
