# Supplementary material for: Correlations between monitor alarm workload and overall workload in Swedish emergency departments: a prospective multicenter cross-sectional study
Source: BMC Emerg Med. 2026 Aug 17;26:224. doi: 10.1186/s12873-026-01733-2 (PMC13488255; doi:10.1186/s12873-026-01733-2)
Supplement: Supplementary file 1 [file 12873_2026_1733_MOESM1_ESM.docx]

Supplements

Questions in the survey

|  | Question in english | Question in Swedish |
| --- | --- | --- |
| 1 | Which emergency department are working at? | Vilken akutmottagning arbetar du på? |
| 2 | Which ED-team have you been assigned to for the last hour? | Vilket team på akutmottagningen har du arbetat den senaste timmen? |
| 3 | How do you perceive your workload in the last hour? | Hur upplever du din arbetsbelastning senaste timmen? |
| 4 | How do you perceive your alarm workload in the last hour? | Hur upplever du din larmbelastning senaste timmen? |
| 5 | In your opinion, has the monitoring equipment been helpful to you in your work in the last hour? | Tycker du att övervakningsutrustningen varit ett hjälpmedel för dig i ditt arbete senaste timmen? |
| 6 | In your opinion, has the monitoring equipment been  disturbing your work in the last hour? | Tycker du att övervakningsutrustningen stört dig i ditt arbete senaste timmen? |
